# Influence of fullerene (C_60_) on soil bacterial communities: aqueous aggregate size and solvent co-introduction effects

**DOI:** 10.1038/srep28069

**Published:** 2016-06-16

**Authors:** Zhong-Hua Tong, Marianne Bischoff, Loring F. Nies, Natalie J. Carroll, Bruce Applegate, Ronald F. Turco

**Affiliations:** 1 CAS Key Laboratory of Urban Pollutant Conversion, Department of Chemistry, University of Science & Technology of China, Hefei, 230026, China; 2 College of Agriculture – Laboratory for Soil Microbiology, Purdue University, West Lafayette, IN 47907, USA; 3 Environmental and Ecological Engineering and the School of Civil Engineering, Purdue University, West Lafayette, IN 47907, USA; 4 Department of Agriculture and Biology Engineering, Purdue University, West Lafayette, IN 47907, USA; 5 Department of Food Science, Purdue University, West Lafayette, IN 47907, USA

## Abstract

Fullerene C_60_ nanoparticles are being used in broad range of applications. It is important to assess their potential impacts in the environment. We evaluated the effects of C_60_ introduced as aqueous suspensions of nC_60_ aggregates of different particle size or via organic solvents on soils with different organic matter contents in this study. Impacts of the application were evaluated by measuring total microbial biomass, metabolic activity and bacterial community structure. Results show that nC_60_ aggregates, introduced as an aqueous suspension, had size-dependent effects on soil bacterial community composition in the low organic matter system, but induced minimal change in the microbial biomass and metabolic activity in soils with both high and low organic matter contents. Fullerene C_60_, co-introduced via an organic solvent, did not influence the response of soil microbes to the organic solvents. Our results suggest that nC_60_ aggregates of smaller size may have negative impact on soil biota and soil organic matter may play a key role in modulating the environmental effect of nanomaterials.

Carbon nanoparticles, such as C_60_, have received a great deal of attention for their unique properties and potential commercial applications^1^. Although hydrophobic in nature, C_60_ will form stable colloidal suspensions in water^2^. This is important because C_60_ may control the availability of pollutants to biological systems^3^,^4^. The C_60_ aggregates in aqueous suspensions (nC_60_) have a crystal structure and can be stable for months, or longer, in low ionic strength solutions^5^, suggesting that nC_60_ aggregates could persist in the environment. This further raises concerns on their potential adverse effects.

Studies in our group have demonstrated fullerene C_60_ when exposed to the environmental matrices such as soil or activated sludge, showed limited effects on microbial activities and community structures^6^,^7^. However, other studies have reported that nC_60_ in water suspensions have strong antibacterial activities^8^ and affect the growth and development of benthic organisms^9^. This inconsistency could be caused by influential factors such as particle size, morphology, surface charge, and environmental conditions^10^,^11^. nC_60_ aggregates of smaller sizes have shown greater antibacterial activity on cultured bacterial cells^8^,^12^. It is still unknown if the size of nC_60_ aggregates is a major factor controlling the response of microorganisms in complex environmental matrices.

Pristine C_60_ is relatively insoluble in water^13^. Organic solvents are used during the manufacturing, processing, application, and handling of C_60_ and C_60_-containing products. Co-exposure with organic solvents has been shown to interfere with xenobiotic metabolic activity in zebrafish larvae^14^, while the biodegradation of polycyclic aromatic hydrocarbons (PAHs) in soils has been observed to increase^15^,^16^. C_60_ nanoparticles are highly hydrophobic (log k_ow_ of 6.67)^13^, suggesting they will tend to absorb to soil. Organic solvents could possibly alter the potential effects of C_60_ in soil by facilitating interactions between C_60_ and soil microorganisms. However, the impact of C_60_ co-introduced in organic solvents on soil microbial processes is largely unknown.

The objective of this study was to examine the response of the soil bacterial community to the introduction of nC_60_ across a range of aggregate sizes, and C_60_ co-introduced in an organic solvent. Different sized nC_60_ aggregates were made by adjusting the mixing rate and ratio of water to a C_60_-saturated tetrahydrofuran (THF) solution. Toluene and THF were selected because: 1) Toluene is a solvent commonly used in many industrial applications, and it also finds use as a solvent to manipulate C_60_ as it can dissolve C_60_ at 2800 mg/L^17^; 2) THF is a water-miscible organic solvent with low boiling point (66 °C) that has been widely used to make water stable dispersions of C_60_ nanoparticles^8^,^18^. The effects of C_60_ on the response of soil microorganisms to toluene and THF were evaluated on soils with different organic matter contents. Results from this study may provide useful information to understand microbial response to C_60_ in environmental matrices.

## Results and Discussion

### nC_60_ aggregate size

In previous reports, differential centrifugation was used to separate nC_60_ aggregates into fractions with different size distributions^19^. Different sizes of nC_60_ aggregates can also be produced by changing the rate of water addition to C_60_-saturated THF during the mixing process^8^, where the particle size decreases as the rate of water addition is increased. In this study, nC_60_ aggregates at an average diameter of 77.8 ± 9.5 nm were generated when 250 mL of water was mixed with 250 mL of C_60_-saturated THF in 2 seconds with fast and vigorous agitation (Supplementary Fig. S1). Aggregates with smaller size were achieved by increasing the volume ratio of water to C_60_-saturated THF, and relatively narrow polydispersity was observed (Supplementary Table S1). Our data show that as the average particle size decreases, more particles with size approaching the mean value were obtained.

### Mineralization activity and microbial biomass

**Figure 1:**
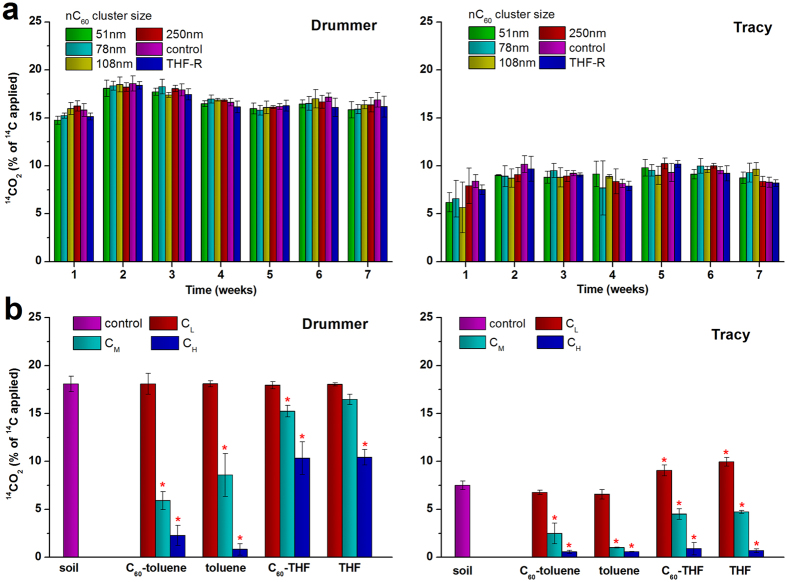
Microbial activity estimated by ^14^CO_2_ released (% of ^14^C applied) 3 hours following ^14^C-labled glucose amendment. (**a**) Soils treated with C_60_ aggregates in aqueous suspensions (nC_60_) of different size, tetrahydrofuran residue (THF-R) and water (control) weekly. (**b**) Soils treated with solvents saturated with or without C_60_ at different doses (denoted as C_L_, C_M_ and C_H_) after a 2-week incubation. Data are mean values ± SD, n = 3, *P < 0.05 compared to the control.

Substrate-induced respiration (SIR) was usually used to estimate soil microbial activity. A readily decomposable substrate was added to soil samples, and the resulting maximal initial respiration response measured over a short time period is used to evaluate the microbial activity in response to toxic materials^20^. In this study, glucose induced respiration in 3 h was used to estimate the mineralization activity of the standing biomass affected by nanomaterial. With repeated application of different sized nC_60_, the levels of ^14^CO_2_ evolved in 3 h after ^14^C-labled glucose addition showed some weekly fluctuations and were higher in the Drummer soil than in the Tracy soil (Fig. 1a), indicating more microbial activity in the Drummer soil, which is typical for soils with higher organic matter^21^. It had been expected that nC_60_ aggregates of smaller particle sizes would have the greatest potential to cause adverse effects on soil microbial activities. However, no significant differences (*P* > 0.05) were observed between the treatments and the control at any time point for either soil, even when the applied nC_60_ had accumulated to 7 μg/g soil at week 7.

Using the same SIR principle, soil response to C_60_ co-introduced with toluene or THF was determined. No significant difference (*P* > 0.05) in ^14^CO_2_ production was observed between samples treated with low doses of toluene, with and without C_60_, and the control for either soil (Fig. 1b). Although THF at low dose did not affect ^14^CO_2_ production in the Drummer soil, they did significantly enhance ^14^CO_2_ production in the Tracy soil. Biodegradation of THF has been reported in several studies^22^,^23^,^24^. Therefore, we suggest that the low dose of THF might stimulate the microbial activity in the Tracy soil. However, it should be noted that the presence of C_60_ did not affect the mineralization activity of soil microbes. For both soils, decreased ^14^CO_2_ production was observed when the solvents were applied at medium and high doses, and toluene shows a stronger inhibitory effect than THF (Fig. 1b) in either soil. Similarly, the presence of C_60_ did not affect ^14^CO_2_ production compared to the non-C_60_ treatment in all cases.

**Figure 2:**
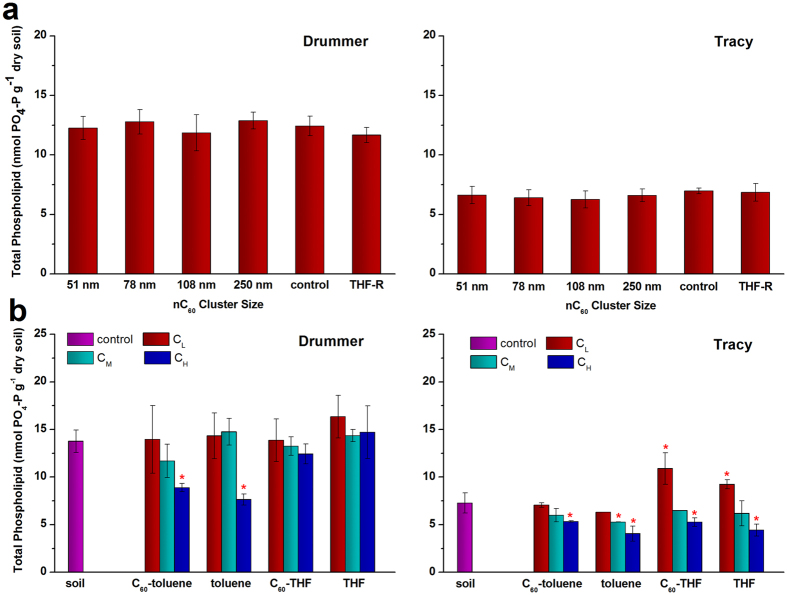
Microbial biomass indicated as total phospholipid-PO_4_. (**a**) Soils treated with tetrahydrofuran residue (THF-R), C_60_ aggregates in aqueous suspensions (nC_60_) of different size compared with the control after a 7-week incubation. (**b**) Soils treated with solvents saturated with or without C_60_ at different doses (denoted as C_L_, C_M_ and C_H_) after a 2-week incubation. Data are mean values ± SD, n = 3, *P < 0.05 compared to the control.

Soil microbial biomass is a measure of the physiologically active part of the soil microbiota that is responsible for critical soil functions. Phospholipids make up cell membrane of living cells; moreover they are an important marker as they change in response to stress and once released to soil, break down rapidly^25^. Therefore, total phospholipid-derived phosphate (PL-PO_4_) is commonly used to estimate soil microbial biomass^26^,^27^. After 7 week incubation for particle size effects, the total microbial biomass estimated PL-PO_4_ concentrations range from 11 to 13 nmol/g for Drummer soil, and 6 to 7 nmol/g for Tracy soil (Fig. 2a). The results of the PL-PO_4_ analysis showed higher microbial biomass in Drummer soil, which has a higher organic matter content, and supports the widely held conclusion that microbial biomass is correlated with soil organic matter contents^28^. However, no significant changes was found (*P* > 0.05) in the size of the microbial biomass as compared to the control soils in either soil. Previous studies have also shown soil respiration and microbial biomass were unaffected by aqueous C_60_ aggregates^29^.

Total microbial biomass was significantly lower (*P* < 0.05) in the Drummer soil treated with a high dose of toluene, and in the Tracy soil with a high dose of either toluene or THF (Fig. 2b). In the two cases, the presence of C_60_ had no effect on the response. At a low dose, THF did not change the level of biomass in the Drummer soil, but increased the size of the biomass in the Tracy soil, where increased microbial activity as estimated by SIR was also evidenced (Fig. 1b). THF has been shown to be biodegradable^22^,^23^,^24^ and our work suggests that THF at low level may be utilized by the soil biomass and the co-introduced C_60_ would not alter this response. Hartmann *et al*.^30^ have also reported that mineralization of sodium acetate by activated sludge were not affected by the presence of nC_60_.

### Bacterial community analysis

**Figure 3:**
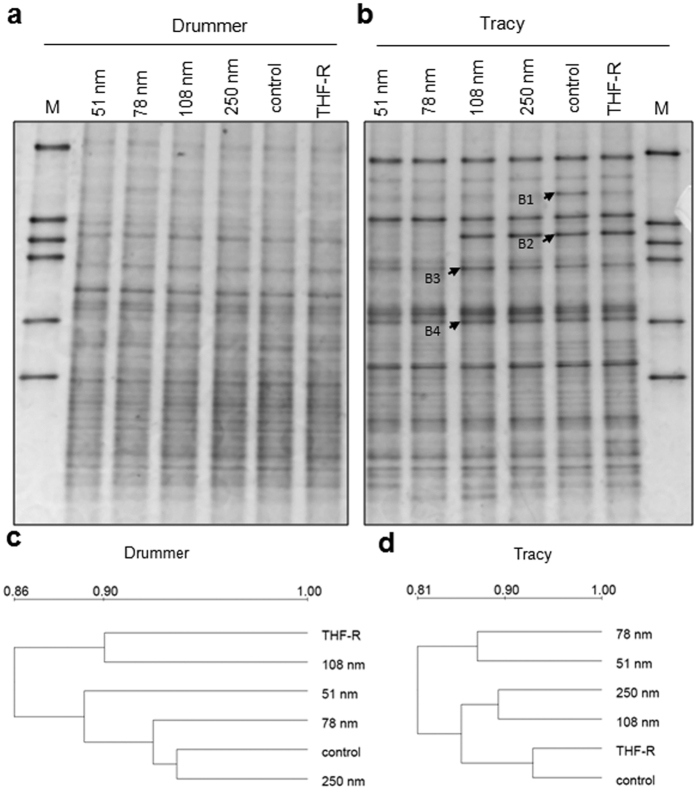
Microbial community analysis of the Drummer and Tracy soil samples treated with C_60_ aggregates in aqueous suspensions (nC_60_) of different size, tetrahydrofuran residue (THF-R), and water (control). (**a,b**) DGGE profiles of 16S rRNA gene fragments. (**c,d**) Dendrograms based on UPGMA cluster analysis showing similarity of the DGGE profiles. Lane M: DGGE marker. Arrows indicate the bands which were extracted from the gels for sequence analysis.

**Figure 4:**
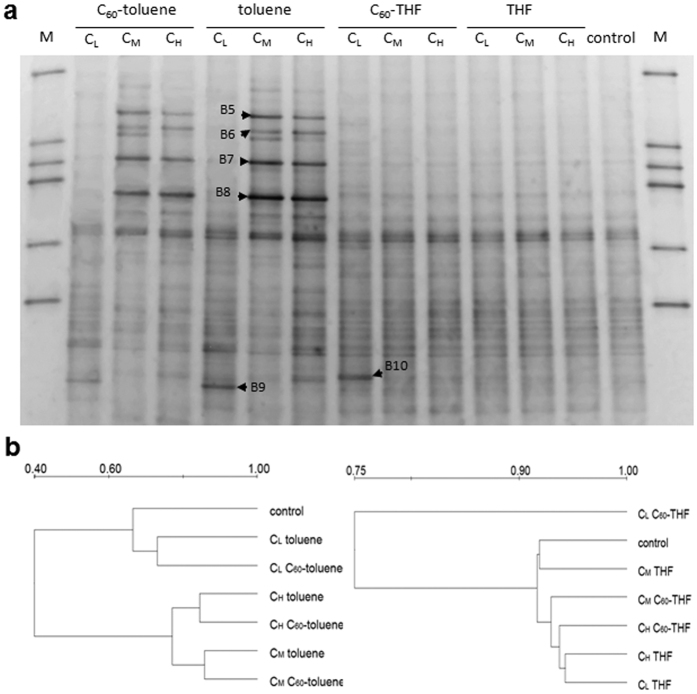
Microbial community analysis for the Drummer soil treated with solvents saturated with or without C_60_ at different doses (denoted as C_L_, C_M_ and C_H_). (**a**) DGGE profiles of 16S rRNA gene fragments. (**b**) Dendrograms based on UPGMA cluster analysis showing similarity of the DGGE profiles. Lane M: DGGE marker. Arrows indicate the bands which were extracted from the gels for sequence analysis.

DGGE patterns of bacterial communities in the treated samples are shown in Figs 3 and 4. For both Drummer and Tracy soils, the DGGE profiles revealed complex banding patterns, indicating a highly diverse bacterial community structure typically found in soils^31^. A number of DGGE bands were unique to each soil suggesting that the soils do support different bacterial populations.

Following the application of nC_60_ aggregates of different sizes to either soil, the DGGE fingerprints yielded around 20 visible bands (Supplementary Fig. S2). The banding patterns of the Drummer soil showed a high degree of similarity, which was also indicated by the dendrogram (Fig. 3a,c). In contrast, a major band (B2) was missing and the intensities of bands B1, B3 and B4 were much lower in the Tracy soil treated with nC_60_ aggregates at 51 and 78 nm (Fig. 3b). Differences in the community structure were also indicated by the dendrogram of DGGE fingerprint. As shown in Fig. 3d, the DGGE profiles were classified into two groups, one with samples treated with the two smaller sized nC_60_ aggregates, and the other group which was further grouped into two clusters with water control and THF residue (THF-R) control samples grouped together. These results demonstrate that nC_60_ aggregates have size-dependent effects on bacterial community structure in soils with low organic matter content. Size-dependent toxicity of nC_60_ aggregates and nanoparticles have been previously reported^10^,^19^,^32^. Sorption of nanomaterials to natural organic matter can strongly reduce their bioavailability and antibacterial activity^33^,^34^.

When the Drummer soil was treated with low doses of toluene either with or without C_60_, few effects on the community profiles were noticed except the intensity of band B9 was enhanced (Fig. 4a). Medium and high doses of toluene produced unique patterns with several strong bands (B5 to B9) regardless of the presence of C_60_. These intensified bands may be the result of a selective enrichment of populations that are tolerant species or capable of toluene degradation. However, THF application had little effect on bacterial community structure except the intensity of band B10 was enhanced (Fig. 4a). These results were also indicated by the cluster analysis (Fig. 4b). The control sample was clustered together with the low dose toluene treatments, and the co-introduced C_60_ soil was grouped together with its respective toluene treatment. In contrast soils receiving low doses of C_60_-saturated THF forms an out-group while others were clustered together with similarity higher than 90%, which could be due to the enhanced band B10.

**Figure 5:**
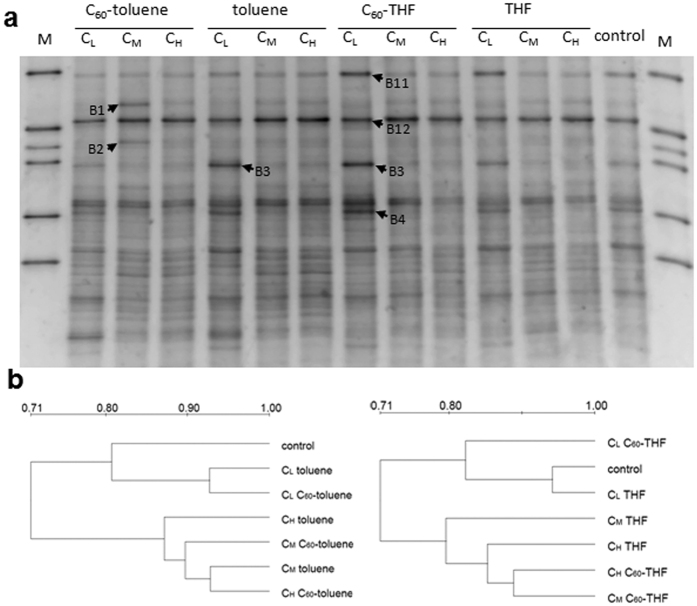
Microbial community analysis for the Tracy soil treated with solvents saturated with or without C_60_ at different doses (denoted as C_L_, C_M_ and C_H_). (**a**) DGGE profiles of 16S rRNA gene fragments. (**b**) Dendrograms based on UPGMA cluster analysis showing similarity of the DGGE profiles. Lane M: DGGE marker. Arrows indicate the bands which were extracted from the gels for sequence analysis.

For the Tracy soil, the medium dose of C_60_-saturated toluene enriched bands B1 and B7 as compared with the control, while the intensity of band B3 was increased with low dose toluene additions (Fig. 5a). These results indicate that bands B1, B3 and B7 may be related to bacteria capable of toluene utilization. The respective dendrogram shows that the DGGE profiles are clustered into two groups, suggesting high doses of toluene can influence the bacterial community (Fig. 5b). Likewise, the samples with medium or high doses of THF were separated from the control and low doses of THF. These results are consistent with DGGE profiles that the intensity of bands B3, B4 and B11 in the Tracy soil were inhibited with THF addition at the two higher doses either with or without C_60_ (Fig. 5a). These findings suggest that co-exposed C_60_ exerts little effect on soil bacterial community structure.

To better understand the changes of bacterial community composition induced by C_60_, the DGGE bands which showed enhanced or reduced intensity were excised and subjected to direct sequence analysis. The closest 16S rRNA gene sequences showing similarities are listed in Table 1. Bands B1, B2, B3 and B4, which are absent or whose intensity increased in samples treated with nC_60_ aggregates of two smaller particle sizes in the Tracy soil, are closely related to uncultured gamma proteobacteria clones (96–100% sequence similarity), indicating they are responsive to nC_60_ aggregate application. Bands B5, B6, B7 and B8 which occurred in Drummer soil treated with medium and high doses of toluene showed greatest similarity to *Bacillus* sp. (97–100%). Bands B9 and B10 show high similarity to *Rhodococcus* sp. (98%) and *Pseudonocardia* sp. (99%), respectively. Isolates of the three groups of bacteria have been previously reported to be capable of toluene degradation^35^,^36^, suggesting the systems may have responded with increases in populations capable of toluene degradation. A similar result has been shown in which bands appeared in DGGE analysis of bacterial communities after acute gamma-irradiation and suggesting surviving bacteria may have thrived and increased their representative populations^37^. Four bands were extracted and sequenced from Tracy soil following solvent applications. Bands B3, B4 and B11 showed high similarity to uncultured gram-negative bacteria, while band B12 showed 100% similarity to *Bacillus benzoevorans*^38^, which is capable of degradation of various aromatic acids and phenols.

## Conclusion

In soil environments, nanomaterials come into contact with both organic matter and salts which create a substantially different microenvironment from test conditions found in water or culture media. Natural organic matter has been shown to disaggregate nC_60_ crystals and change the particle size^39^,^40^. On the other hand, salts at high concentrations in soil, especially calcium, will cause precipitation of the C_60_ from the aqueous suspension^39^, and as a result, the actual dose of nC_60_ will be much lower. Nonetheless, in the present study, the two smallest sized nC_60_ aggregates shifted the bacterial community composition in the soil with low organic matter content, suggesting that they are toxic to soil biota. Previous research has attributed the toxic effects to THF decomposition products rather than to nC_60_ alone^41^,^42^. However, our results demonstrate little adverse effects for the residual THF control in both soils. The highly complex nature of the soil system may have masked the adverse effects of any THF derived chemicals applied in trace amounts. Although organic solvents showed concentration-dependent effects on soil microbial activity and community structure, the co-introduced C_60_ have minimal effect even when their doses were much higher than that of nC_60_ aggregates. Their adverse effects might be shielded by large amount of carrier solvents. Overall, our results suggest that nC_60_ aggregates of smaller size may have negative impact on soil biota and soil organic matter may play a key role in modulating the environmental effect of nanomaterials.

## Methods

### Aqueous C_60_ preparation

Aqueous dispersions of C_60_ (nC_60_) aggregates were prepared using a procedure from Fortner *et al*.^8^ modified as follows. Approximately 12 mg of C_60_ (99.5%, Sigma-Aldrich, St. Louis, MO ) were added to 500 mL of newly distilled THF (HPLC grade, 99.9%, Fisher Scientific) and stirred for 24 h in the dark at ambient temperature. Upon saturation, the solution was vacuum filtered through a 0.22 μm membrane. Then 250 mL of distilled water was added to 250 mL of C_60_-saturated THF solution with vigorous stirring. Changing the rate of water addition (1875, 1000, 250, 120, 30, 20 mL/min) will result in aggregates of different sizes. The mixed solutions were then gently heated using a rotary evaporator (Büchi Rotovap) to remove THF as described in Fortner *et al*.^8^, and concentrated as needed. A THF-R was generated in the same way but without adding C_60_. To maintain sterility, all containers, stir bars and distilled water were autoclaved. Concentrations of nC_60_ were measured spectrophotometrically^6^ and the resulting stock solutions were at 25–45 μg/mL. The average diameters of nC_60_ aggregates were determined by dynamic light scattering using a DynaPro-99 (Protein Solutions).

### Soil sampling and microcosm experiments

The surface soils used in this study were the same as described previously^43^. The Drummer soil (fine-silty, mixed, superactive, mesic Typic Endoaquoll, 3.6% organic matter) was collected from the Purdue Agriculture Research Center, West Lafayette, IN. The Tracy soil (coarse-loamy, mixed, active, mesic Ultic Hapludalfs, 1.5% organic matter) was collected from the Pinney-Purdue Agriculture Center near Wanatah, IN. The soil properties were listed in Supplementary Table S2. Soils were sieved with 4 mm mesh, homogeneously mixed and stored in closed containers at room temperature.

To study the nC_60_ aggregate size effects, soil microcosm was constructed in a 250-mL screw-top jar containing 100 g of soil (dry weight). Soil moisture was adjusted with sterile distilled water to field capacity, which is 29.2% for the Drummer soil, and 15.2% for the Tracy soil. The microcosms were preincubated at 23 °C for 4 days. Then, nC_60_ aggregates with average diameter 51, 78, 108 or 250 nm were added into different microcosms at 1 μg/g soil, respectively, and thoroughly mixed with a spatula. Sterile distilled water and THF-R were added as controls. The treatments were reapplied weekly and done in triplicate at 23 °C for 7 weeks. SIR was conducted on 10 g (dry weight) subsamples taken from the microcosms weekly before reapplication of treatments. After 7 weeks, SIR, microbial biomass analysis and bacterial community profiling by PCR-DGGE were done.

For solvent co-introduction study, 50 g of soil (dry weight) was added into a 125-mL screw-top jar. Solvent saturated with C_60_ was generated by adding excess granular C_60_ (99.5%) to THF or toluene (HPLC, 99.9%, Fisher Scientific). The solutions were shaken for 24 h at 125 rpm on a gyratory shaker at room temperature. Undissolved C_60_ was removed with a 0.22 μm nylon membrane filter. After 4 days of preincubation, soil samples were exposed to C_60_-saturated solvents at 1, 5 and 10 mg/g soil, denoted as C_L_, C_M_ and C_H_. The highest dose of the solvent was less than 10% of the soil field capacity to minimize toxic effects of the solvents. Because of the different solubility of C_60_ in toluene and THF, C_60_ was applied to soil samples at 0.01, 0.05 and 0.1 μg/g via THF, and 3.24, 16.18 and 32.37 μg/g via toluene, respectively, representing low to high doses of C_60_ (Table 2). Treatments with same amount of solvents and water controls were also included. All treatments were conducted at 23 °C in triplicate. Our preliminary study showed prolonged incubation with repeated application of low doses of solvents will result in microbial utilization of the solvents. Therefore, a two-week experiment with a one-time dosing was conducted. After two weeks, SIR, microbial biomass analysis and bacterial community profiling by PCR-DGGE were done.

### Microbial biomass and activity assessment

After completion of the nC_60_ aggregate study (7 weeks) and the solvent co-introduction study (2 weeks), subsamples from the microcosms were sampled and freeze-dried. Microbial biomass was determined by measuring PL-PO_4_ of the lyophilized soil sample. The phospholipid was extracted and the PL-PO_4_ was measured using a previously described colorimetric method^44^.

The ability of the soil biomass to mineralize glucose was measured weekly for the 7-week nC_60_ aggregate study and at the end of solvent co-introduction study, by treating subsoil samples from each microcosm with D-glucose-UL-^14^C (specific activity = 264 mCi/mmol, Sigma-Aldrich, St. Louis, MO) to create an SIR response using a procedure as previously described^43^.

### Soil bacterial community analysis using PCR-DGGE

At the indicated time, a 0.5-g soil sample from each microcosm was removed for DNA extraction. The genomic DNA was isolated using the FastDNA^®^ SPIN kit for soil (MP Biomedical, Solon, OH) according to the manufacturer’s instructions. The quantity and quality of isolated DNA were determined using a Nanodrop^®^ ND-1000 Spectrophotometer (NanoDrop Technologies, Wilmington, DE). DNA extracts were also inspected by electrophoresis in 0.7% agarose gels with ethidium bromide staining.

PCR was performed using the universal primers for bacteria F338-GC and R534 as described by Muyzer *et al*.^45^ in a thermocycler (Eppendorf North America, Westbury, NY) using the following modified protocol: 94 °C for 5 min, followed by 30 cycles of 94 °C for 0.5 min, 55 °C for 0.5 min and 72 °C for 0.5 min, then a final extension step at 72 °C for 10 min. The resulting PCR products were analyzed with 1% agarose gel electrophoresis. The triplicate DGGE profiles were highly similar, and thus the triplicate PCR products were pooled for DGGE analysis.

PCR products were loaded on an 8% polyacrylamide gel with a denaturant gradient ranging from 35 to 70% and run at 60 °C and 75V for 16 h on a DCode system (Bio-Rad Laboratories, Hercules, CA). Bands were stained for 20 min in 1X TAE containing a 1:5,000 dilution of SYBR green I dye (Cambrex Bioscience, Walkersville, MD) and visualized under UV transillumination using the Kodak Imaging Station (Eastman Kodak Co., Rochester, NY). Band patterns and average intensities of the bands were analyzed using Quantity One, version 4.6.8 (Bio-Rad Laboratories). Profile similarity was analyzed using the unweighted pair group method with arithmetic mean (UPGMA).

The DGGE bands whose intensity appeared to be either enhanced or reduced were excised and suspended in 100 μL nuclease-free water overnight. The eluted DNA fragments were reamplified and run on the DGGE gel to ensure purity and correct mobility. Products showing one distinct band with correct mobility were further amplified with the primers for DGGE analysis but without a GC clamp. The PCR products were purified using a QIAquick gel extraction kit (QIAGEN, Valencia, CA) and submitted for sequencing at Purdue University’s Genomics Facility. The sequence data of DGGE bands were compared to sequences in GenBank database of the National Center for Biotechnology Information (NCBI) using the BLASTn program, and have been deposited in the GenBank database (Table 1) under accession numbers GQ470401 to GQ470410, KP204446, and KP217807.

## Additional Information

**How to cite this article**: Tong, Z.-H. *et al*. Influence of fullerene (C_60_) on soil bacterial communities: aqueous aggregate size and solvent co-introduction effects. *Sci. Rep.* **6**, 28069; doi: 10.1038/srep28069 (2016).

## Supplementary Material

Supplementary Information

## Figures and Tables

**Table 1:** Sequence similarities of recovered DGGE Bands.

Bands (GenBank accession number)	Most closely related database entry (GenBank accession number)	Similarity
B1 (KP217807)	Uncultured *Coxiellaceae* bacterium clone (JX576004)	96%
B2 (KP204446)	Uncultured gamma proteobacterium clone (LN567544)	98%
B3 (GQ470409)	Uncultured *Rhodanobacter* sp. clone (DQ145582)	100%
B4 (GQ470410)	Uncultured gamma proteobacterium clone (EF072022)	97%
B5 (GQ470401)	*Bacillus megaterium* (EU723823)	97%
B6 (GQ470402)	*Bacillus* sp. 05 (EU399813)	98%
B7 (GQ470403)	*Bacillus* sp. C-17 (EU809476)	100%
B8 (GQ470404)	*Bacillus* sp. RM1A (EF765626)	97%
B9 (GQ470405)	*Rhodococcus* sp. VC-YC6630CNS139 (EU734599)	98%
B10 (GQ470406)	*Pseudonocardia* sp. (DQ448726)	99%
B11 (GQ470407)	Uncultured *Flavobacteria* bacterium clone (EF650874)	100%
B12 (GQ470408)	*Bacillus benzoevorans* (EU744622)	100%

**Table 2:** Dose of organic solvents and C_60_ applied to soil.

Dose	C_60_-toluene		C_60_-THF	
	C_60_ (μg/g)	toluene (mg/g)	C_60_ (μg/g)	THF (mg/g)
C_L_	3.24	1	0.01	1
C_M_	16.18	5	0.05	5
C_H_	32.37	10	0.1	10
