# Supplementary material for: Influence of fullerene (C60) on soil bacterial communities: aqueous aggregate size and solvent co-introduction effects
Source: Sci Rep. 2016 Jun 16;6:28069. doi: 10.1038/srep28069 (PMC4910098; doi:10.1038/srep28069)
Supplement: Supplementary Information [file srep28069-s1.pdf]

Supplementary Information for:

Influence of fullerene (C_{60}) on soil bacterial communities: aqueous aggregate size and solvent co-introduction effects

Zhonghua Tong [1,2], Marianne Bischoff [2], Loring F. Nies[3], Natalie J. Carroll[4], Bruce Applegate[5], Ronald F. Turco[2]

[1]CAS Key Laboratory of Urban Pollutant Conversion, Department of Chemistry, University of Science & Technology of China, Hefei, 230026, China.
[2]College of Agriculture – Laboratory for Soil Microbiology, Purdue University, West Lafayette, IN 47907, USA.
[3]Environmental and Ecological Engineering and the School of Civil Engineering, Purdue University, West Lafayette, IN 47907, USA.
[4]Department of Agriculture and Biology Engineering, Purdue University, West Lafayette, IN 47907, USA.
[5]Department of Food Science, Purdue University, West Lafayette, IN 47907, USA.

[*]Corresponding author:

Prof. Ronald F. Turco, phone: 765 494 8077; fax: 765 496 2926; e-mail: rturco@purdue.edu

Table S1. Average particle sizes of aqueous nC_{60} aggregate.

Average size (nm)	50.6	77.8	108.1	148.3	250.2
Standard deviation	9.8	9.5	10.5	41.4	97.9

Table S2. Properties of soils used in this study.

Soil	Soil texture	Organic matter (%)	Sand (%)	Silt (%)	Clay (%)	pH
Drummer	Silty clay loam	3.6	16.8	52.4	30.8	6.9
	Taxonomic class: fine-silty, mixed, superactive, mesic Typic Endoaquolls					
Tracy	Sandy loam	1.5	55.2	36.8	8.0	6.1
	Taxonomic class: coarse-loamy, mixed, active, mesic Ultic Hapludalfs					

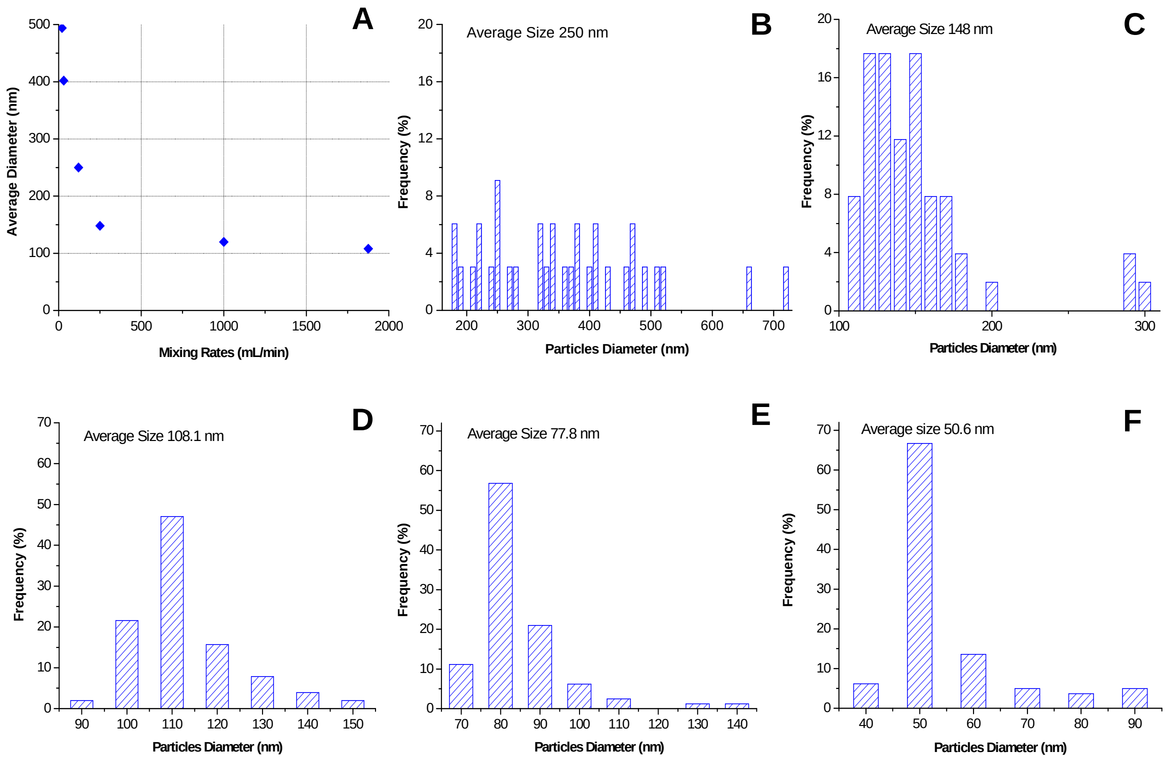

Figure S1. Effect of the rate of water addition on nC_{60} size formation. (A) Average aggregate size as a function of the rate of water addition. (B) Mixing at 120 mL/min. (C) Mixing at 250 mL/min. (D) Mixing at 1875 mL/min. (E) 250 mL water added to 250 mL THF with dissolved C_{60} in 2 seconds. (F) 600 mL water added to 35 mL THF with dissolved C_{60} in 2 seconds.

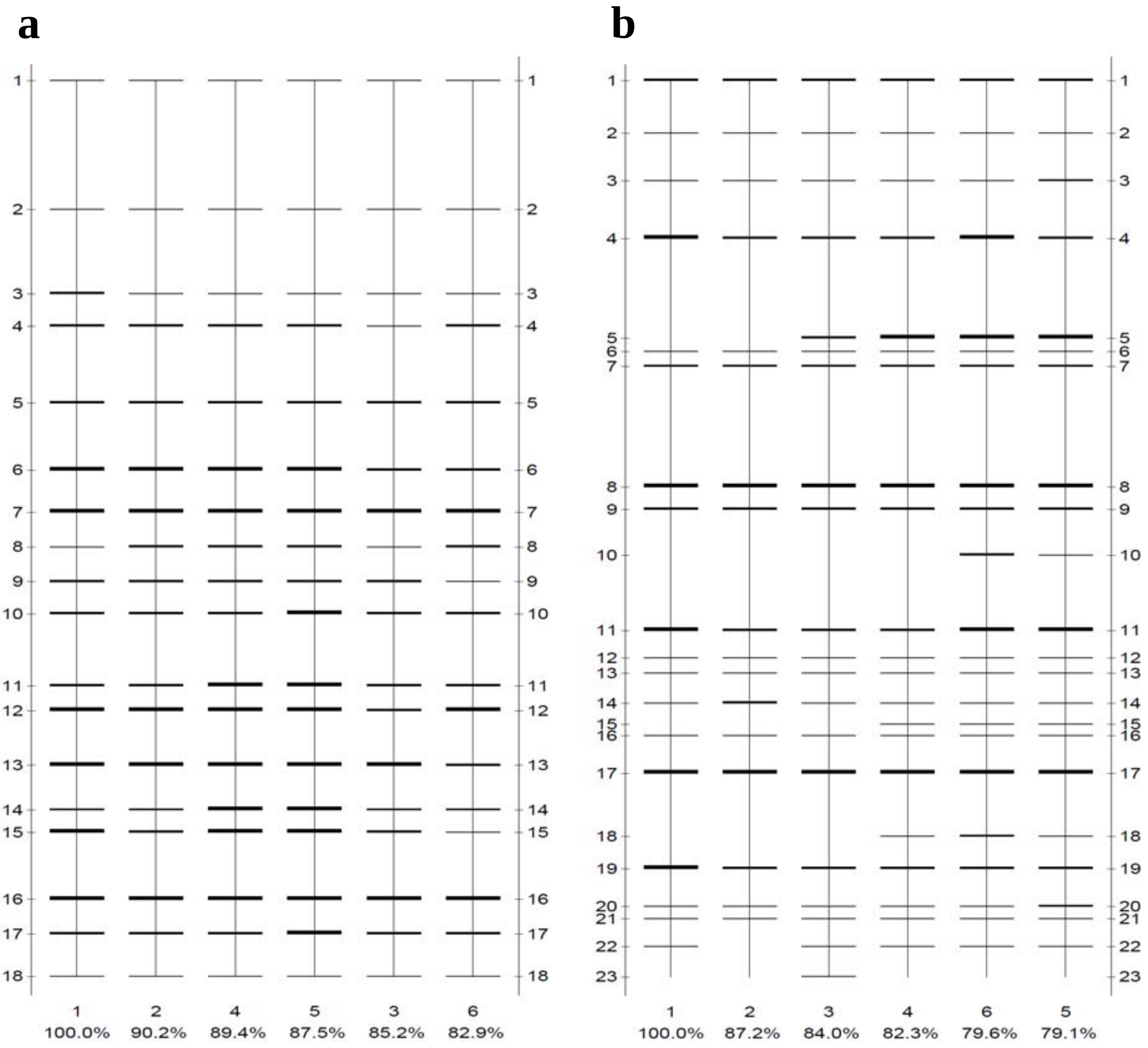

Figure S2. Schematic drawings of bacterial DGGE profiles of samples treated with nC_{60} of different size, THF residue (THF-R), and water (control). (a) Drummer soil; (b) Tracy soil. Lane 1, 51 nm; Lane 2, 78 nm; Lane 3, 108 nm; Lane 4, 250 nm; Lane 5, control; Lane 6, THF-R.

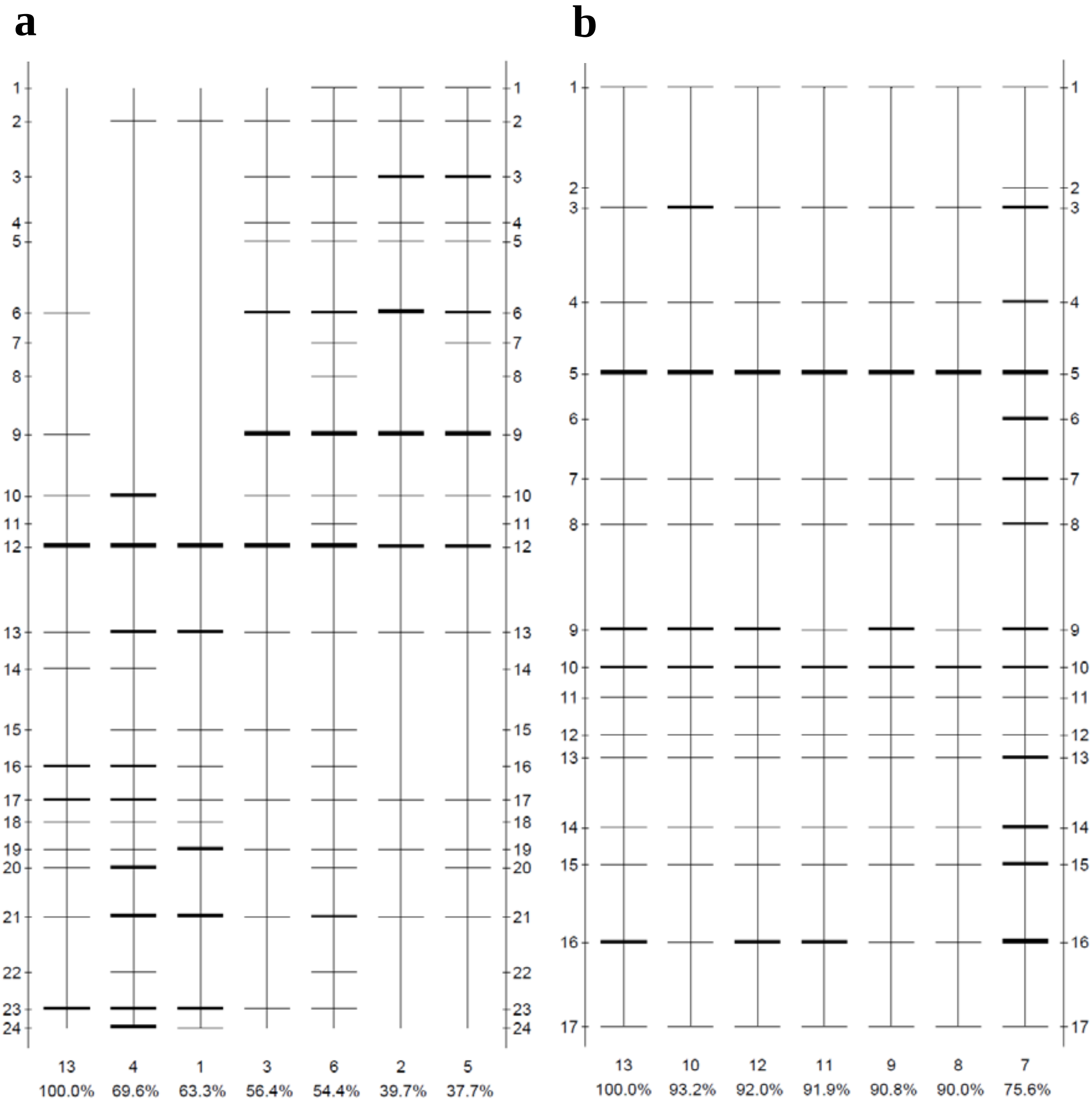

Figure S3. Schematic drawings of bacterial DGGE profiles for the Drummer soil treated with solvents saturated with or without C_{60} at different doses (denoted as C_L, C_M and C_H). (a) toluene; (b) THF. Lane 1, C_L/C_{60}-toluene; Lane 2, C_M/C_{60}-toluene; Lane 3, C_H/C_{60}-toluene; Lane 4, C_L/toluene; Lane 5, C_M/toluene; Lane 6, C_H/toluene; Lane 7, C_L/C_{60}-THF; Lane 8, C_M/C_{60}-THF; Lane 9, C_H/C_{60}-THF; Lane 10, C_L/THF; Lane 11, C_M/THF; Lane 12, C_H/THF; Lane 13, control.

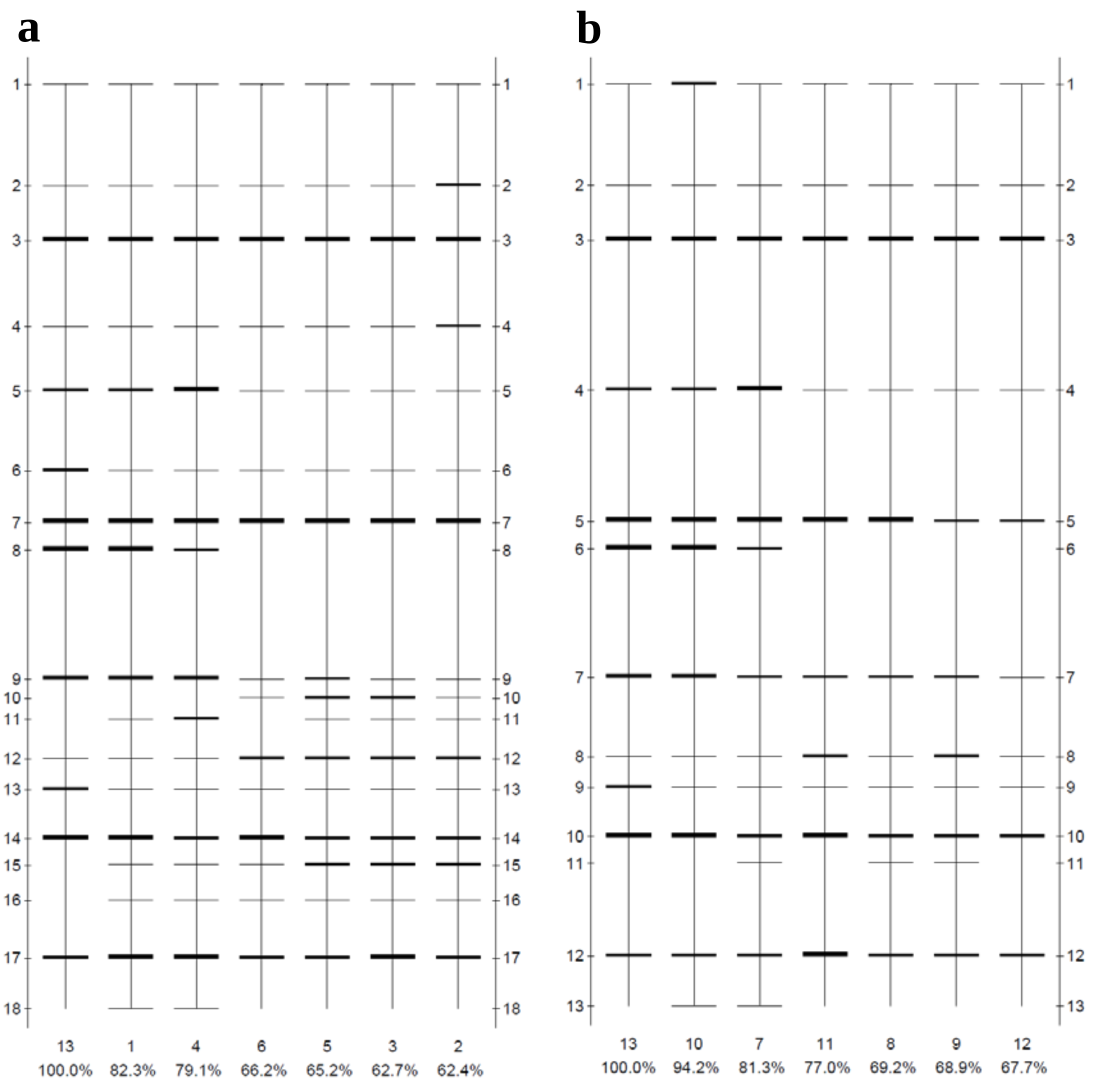

Figure S4. Schematic drawings of bacterial DGGE profiles for the Tracy soil treated with solvents saturated with or without C_{60} at different doses (denoted as C_L, C_M and C_H). (a) toluene; (b) THF. Lane 1, C_L/C_{60}-toluene; Lane 2, C_M/C_{60}-toluene; Lane 3, C_H/C_{60}-toluene; Lane 4, C_L/toluene; Lane 5, C_M/toluene; Lane 6, C_H/toluene; Lane 7, C_L/C_{60}-THF; Lane 8, C_M/C_{60}-THF; Lane 9, C_H/C_{60}-THF; Lane 10, C_L/THF; Lane 11, C_M/THF; Lane 12, C_H/THF; Lane 13, control.
